# RE-EMPLOYMENT MANDATE IN SINGAPORE: EMPLOYMENT AND HEALTH OUTCOMES

**DOI:** 10.1093/geroni/igac059.1073

**Published:** 2022-12-20

**Authors:** Ngee Choon Chia, Cynthia Chen, Jemima Koh, Kia Yee Lim, Yuet Yan Tsoi

**Affiliations:** National University of Singapore, Singapore, Singapore; National University of Singapore, Singapore, Singapore; National University of Singapore, Singapore, Singapore; National University of Singapore, Singapore, Singapore; National University of Singapore, Singapore, Singapore

## Abstract

Older workers can be a crucial resource to meet manpower needs for aging society. This is especially so for countries where older adults are expected to live longer and healthier. In 2012, Singapore implemented the Retirement and Re-employment Act (RRA) which obliged employers to offer re-employment to eligible workers. With RRA, mature workers have the flexibility to work beyond retirement age. Using the Retirement and Health Study (RHS) data and regression discontinuity design, we find that, after adjusting for education, marital status, housing asset and expenditure, the re-employment mandate helps delay retirement in the sample by 8.7%, with a larger impact of 9.1% among the males, as compared to the females of 7.3%. More non-retirees reported that they have very good or excellent health compared to retirees. Retirement leads to increase in healthcare utilization. Does the work environment affect how one responds to retirement option? We observe a correlation between post-retirement wellbeing and stress at work. Our study also suggests that mandatory re-employment offers are less effective in encouraging those in physically demanding jobs to continue working. Re-employment mandate helped raise the employment rate for workers in high-routine, high-manual jobs by 6.1 per cent. These would include craft workers and trade workers. It raised the employment rate for workers in highly routine and low-manual occupations by 26.9 per cent. These include workers in the hospitality, retail and services sectors. In sum, the mandate together with other inclusive work policies have been useful to mitigate impacts of societal ageing in Singapore.

